# Sex hormones regulate the sexual dimorphism of the lung resident immune milieu

**DOI:** 10.1038/s41598-025-15941-6

**Published:** 2025-08-31

**Authors:** Ioannis Belios, Tao Zhang, Christopher Urbschat, Jun Oh, Wolfgang Jungraithmayr, Samuel Huber, Petra C. Arck, Anastasios D. Giannou, Dimitra E. Zazara

**Affiliations:** 1 Division for Experimental Feto-Maternal Medicine, Department of Obstetrics and Fetal Medicine, University Medical Center Hamburg-Eppendorf (UKE), Hamburg, Germany; 2 Section of Molecular Immunology and Gastroenterology, I. Department of Medicine, UKE, Hamburg, Germany; 3 University Children’s Hospital, UKE, Hamburg, Germany; 4 Division of Thoracic Surgery, Rostock University Medical Center, Rostock, Germany; 5 Department of Thoracic Surgery, University Hospital Zurich, Zurich, Switzerland; 6 Department of Thoracic Surgery, Faculty of Medicine, Medical Center-University of Freiburg, Faculty of Medicine, University of Freiburg, Freiburg, Germany; 7 Hamburg Center for Translational Immunology, UKE, Hamburg, Germany; 8 Department of General, Visceral and Thoracic Surgery, UKE, Hamburg, Germany; 9 Division for Experimental Feto-Maternal Medicine, Department of Obstetrics and Fetal Medicine and University Children’s Hospital, University Medical Center Hamburg-Eppendorf, Martinistr. 52, 20246 Hamburg, Germany

**Keywords:** Tissue-resident immunity, Lung, Sexual dimorphism, Testosterone, Immunology, Innate immunity

## Abstract

A strong sex-bias characterizes many respiratory immune diseases and has been attributed to sexually dimorphic immune responses. However, the role of lung-resident immunity in this context remains elusive. Here, we thoroughly characterized the lung-resident immune landscape in male and female mice, with a special focus on sex hormone effects in this context. Androgens were found to exert the strongest effects, markedly impacting B cells and neutrophils in both male and female lungs. Castrated males exhibited increased, while testosterone-treated females and males decreased lung-residing B cells. Testosterone supplementation of castrated males and females resulted in increased lung-residing neutrophils. Sex-mismatched orthotopic lung transplantation further supported these findings, since lungs isolated from female donors exhibited reduced tissue-residing B cells after their transplantation into male recipients. For the remaining lung-resident immune cell populations, sex differences were observed at the level of cell frequencies, with male lungs exhibiting higher frequencies of alveolar macrophages and lower frequencies of lung-resident dendritic cells and CD4⁺ tissue-resident memory T cells. Castration reversed some of these findings. Our findings highlight that the sexual dimorphism of the lung-resident immune landscape is modulated by sex hormones and especially androgens, thereby providing insights into the sex-specific manifestation of respiratory immune diseases.

**Supplementary Information:**

The online version contains supplementary material available at 10.1038/s41598-025-15941-6.

## Introduction

The prevalence and manifestation of respiratory immune diseases often differ in a sex-specific manner^1^. For example, adult women seem to suffer more frequently from asthma and chronic obstructive pulmonary disease (COPD), while men are more susceptible to respiratory infections such as pneumococcal pneumonia^2–5^. Since respiratory diseases are one of the top three global causes of death, according to the World’s Health Organization (WHO)^6^, identifying the molecular and cellular pathways leading to these sex differences is essential.

Mounting evidence suggests that sex steroids significantly contribute to the sex bias observed in the context of respiratory diseases^7^. A well-studied disease supporting this theory is asthma. Asthma is more prevalent in males until puberty, while in adolescence and adulthood this trend reverses with females being mostly affected^8^. Furthermore, alterations in the severity of asthma observed during both the different phases of the menstrual cycle^9^ and pregnancy^10^ further support a role of sex hormones in disease manifestation and course. Based on the differential sex bias that is observed in asthma in different stages of life, testosterone is considered to reduce the risk of asthma, while estrogens are related to higher risks^11^.

Recent studies highlight an important role of tissue-resident immunity in sustaining lung homeostasis and—when disrupted—in the pathogenesis of respiratory diseases^12^. Tissue-resident immunity comprises a network of non-circulating innate and adaptive immune cells, which are responsible for tissue immune surveillance and act as first responders upon pathogen intrusion^12^. Key lung-resident immune cell populations include the alveolar macrophages (AM), the dendritic cells (DCs), the innate lymphoid cells (ILCs), and the tissue-resident memory T cells (TRMs)^12^. Indeed, depletion of one of these populations in mouse models of various respiratory infections leads to higher disease burden and worse outcomes^13,14^.

Despite rising evidence demonstrating the importance of tissue-resident immunity for respiratory health, potential sex differences in this immune compartment and its role in the sex bias of respiratory diseases remain elusive. Since tissue-resident immunity is crucially involved in respiratory disease pathogenesis, we here hypothesized that lung-resident immunity differs in a sex-specific manner already before disease manifestation and can be modulated by sex hormones thereby contributing to sex-specific disease susceptibility and course. Using hormone manipulation approaches, we thoroughly characterized the sexual dimorphism of lung-resident immunity and the role of sex hormones in this context.

## Results

### Sex-specific differences in innate and adaptive tissue-resident immunity in the naïve mouse lung

**Fig. 1:**
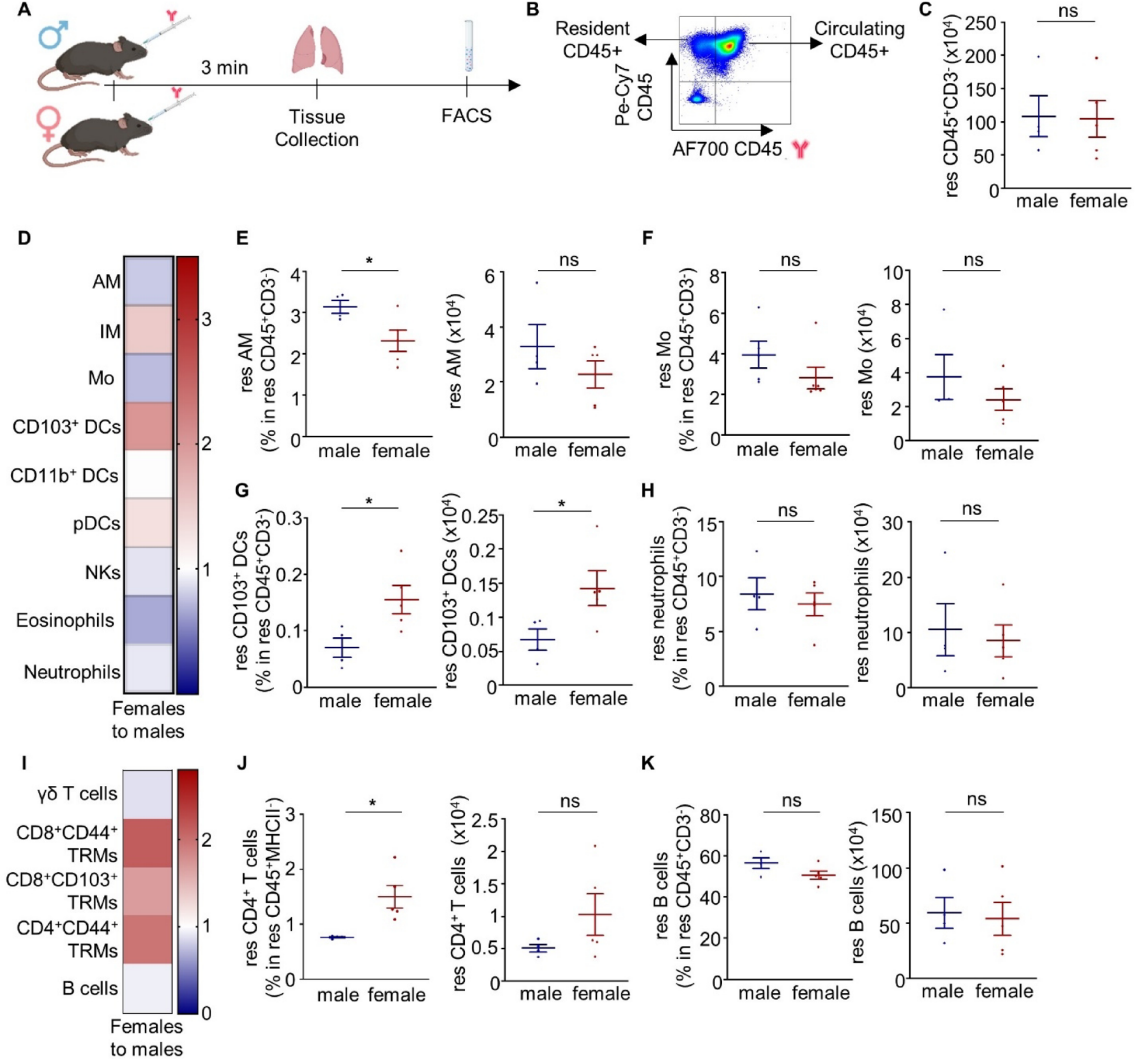
Sex differences in mouse lung-resident immune landscape. **(A)** Schematic view of the experimental setup (created with Biorender). **(B)** Flow cytometry-based distinction of lung-resident and circulating CD45^+^ cells. **(C)** Number of resident CD45^+^CD3^-^ cells in male and female lungs. **(D)** Heatmap depicting the frequency of innate immune cells residing in the female lung normalised to the frequency of resident innate immune cells of the male lung. **(E–H)** Frequency in resident CD45^+^CD3^−^ cells and number of resident **(E)** alveolar macrophages, **(F)** monocytes, **(G)** CD103^+^ DCs, and **(H)** neutrophils in male and female mouse lungs. **(I)** Heatmap depicting the frequency of adaptive resident immune cells in the female lung normalised to the frequency of the respective populations in the male lung. **(J)** Frequency in resident CD45^+^MHCII^−^ cells and number of resident CD4^+^ T cells in male and female mouse lungs. **(K)** Frequency in resident CD45^+^CD3^−^ cells and number of resident B cells in male and female mouse lungs. *n* = 4–5 mice per group. Each experiment was repeated twice. Data are shown as mean ± SEM. *: *p* ≤ 0.05, **: *p* ≤ 0.01 as assessed by Mann-Whitney test. Non-significant differences (*p* > 0.05) are stated as ns. res: resident; AM: alveolar macrophages; IM: interstitial macrophages; Mo: monocytes; DCs: dendritic cells; NKs: natural killer cells; TRMs: tissue-resident memory T cells.

To identify potential sex differences in lung-resident immunity, adult male and female naïve C57BL/6 mice received a CD45:AF700 antibody intravenously 3 min prior to sacrificing (Fig. 1A). This intravascular staining approach allowed a distinction between circulating leukocytes within the vessels supplying the lung (referred to as circulating cells), and leukocytes outside the blood circulation residing in the lung parenchyma (referred to as resident cells)^12,15^ (Fig. 1B, Supplementary Figs. 1 and 2). Interestingly, several innate and adaptive lung-resident immune cell populations were found to differ between male and female mice (Fig. 1C–K, Supplementary Fig. 3). Specifically, increased frequencies of alveolar macrophages were found residing in the lungs of male compared to female mice, while the same trend was observed concerning the number of these cells (Fig. 1E). On the other hand, female lungs exhibited higher tissue-resident CD103^+^ dendritic cells (DCs), both in numbers and frequency, compared to the male ones (Fig. 1G). Similarly, increased frequency of CD4^+^ TRM cells were observed in female lungs compared to male ones (Fig. 1J). Sex differences in lung innate lymphoid cells (ILCs), as well as the effect of sex hormones on them have been thoroughly described before^16^. In accordance with published evidence, we observed a higher frequency of ILC2s (Supplementary Fig. 4J) within the total CD45⁺ population in females compared to males. However, this difference was not evident regarding the lung-resident ILC2 or the circulating subset separately (Supplementary Fig. 4D, G). No differences in ILC1 or ILC3 were detected (Supplementary Fig. 4C).

### Androgen level modulation impacts lung-resident immunity in male mice

**Fig. 2:**
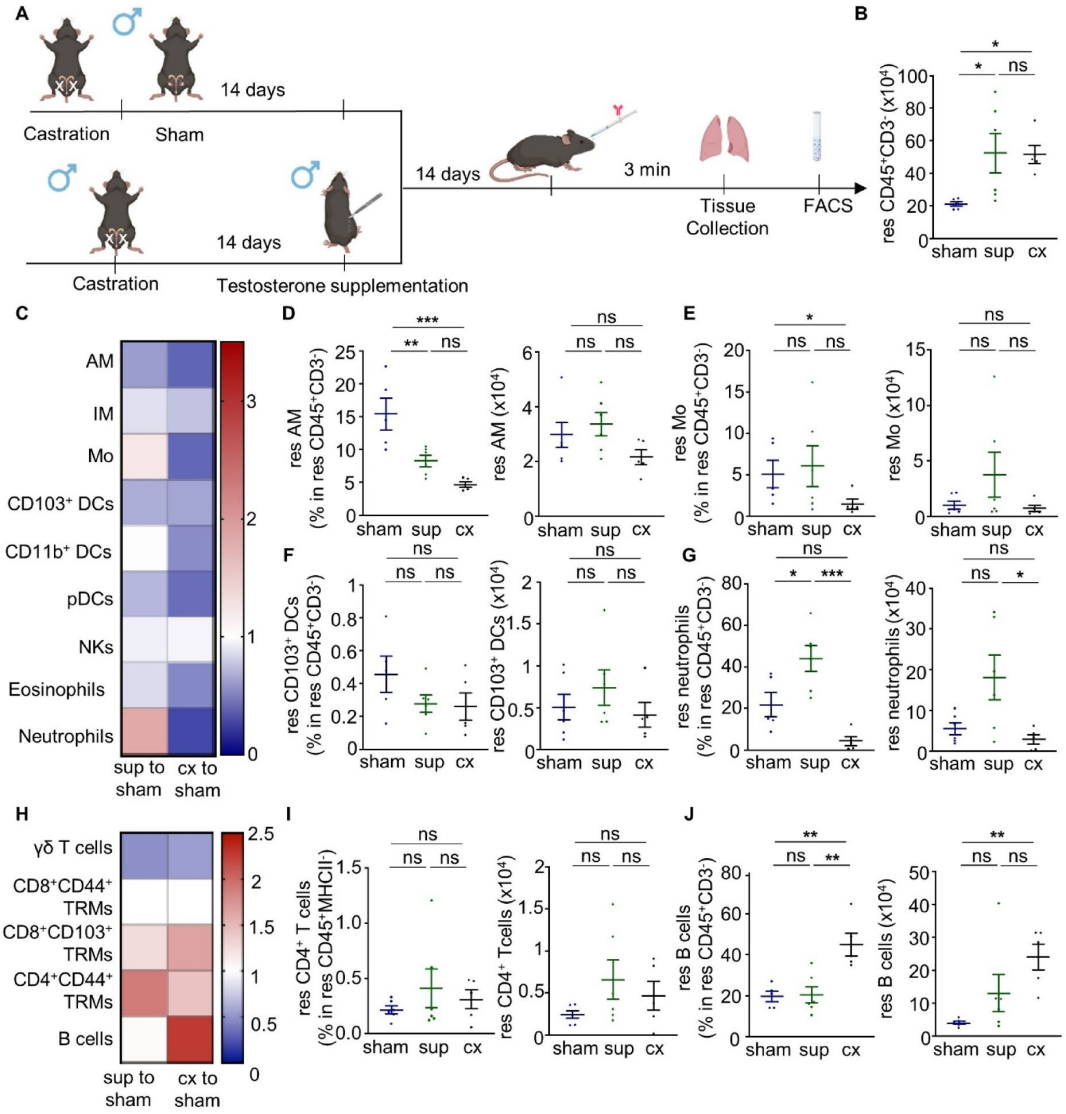
Testosterone affects lung-resident immunity in male mice. **(A)** Schematic view of the experimental setup (created with Biorender). **(B)** Number of resident CD45^+^CD3^-^ cells in the lungs of sham operated, castrated and then supplemented with testosterone (sup), as well as castrated (cx) male mice. **(C)** Heatmap depicting the frequency of innate resident immune cells in the lungs of castrated (cx) male mice normalised to the frequency of innate resident immune cells in the lungs of sham operated (sham) male mice and also of castrated mice and then supplemented with testosterone (sup) normalised again to the frequency of innate resident immune cells in the lungs of sham operated (sham) male mice. **(D**–**G)** Frequency in resident CD45^+^CD3^−^ cells and number of resident **(D)** alveolar macrophages, **(E)** monocytes, **(F)** CD103^+^ DCs, and **(G)** neutrophils. **(H)** Heatmap depicting the frequency of adaptive immune cells residing in the lungs of castrated male mice normalised to the frequency of adaptive immune cells residing in the lungs of sham operated male mice and also of castrated mice and then supplemented with testosterone (sup) normalised again to the frequency of adaptive immune cells residing in the lungs of sham operated male mice. **(I)** Frequency in resident CD45^+^MHCII^−^ cells and number of resident CD4^+^ T cells. **(J)** Frequency in resident CD45^+^CD3^−^ cells and number of resident B cells. *n* = 5–6 mice per group. Each experiment was repeated twice. Data are shown as mean ± SEM. *: *p* ≤ 0.05, **: *p* ≤ 0.01 as assessed by Mann-Whitney test. Non-significant differences (*p* > 0.05) are stated as ns. res: resident; AM: alveolar macrophages; IM: interstitial macrophages; Mo: monocytes; DCs: dendritic cells; pDCs: plasmacytoid DCs; NKs: natural killer cells; TRMs: tissue-resident memory T cells.

Given the well-documented effect of sex hormones on circulating immunity, we next sought to investigate the potential impact of androgens on lung-resident immunity using castration in male mice. 12-week-old male C57BL/6 mice underwent castration, while control male mice of the same age were sham operated, as described below. 28 days after the operation, intravascular leukocyte staining was applied, the mice were subsequently sacrificed, and their lungs were collected (Fig. 2A–C). To further investigate the effect of androgens on lung-resident immunity, a group of castrated male mice received testosterone supplementation 14 days after castration and were sacrificed 14 days later (Fig. 2A). Of note, lung neutrophils and B cells were most profoundly affected by androgen level modulation. Specifically, a higher frequency and number of resident B cells was observed in the lungs of male castrated mice (Fig. 2J), while testosterone supplementation of castrated male mice resulted in lung-residing B cell reduction, which then reached the levels observed in sham operated control mice (Fig. 2J). Moreover, testosterone supplementation of castrated male mice was associated with highly increased lung-residing neutrophils, as compared to both sham operated and castrated male mice (Fig. 2G). Significantly decreased frequencies, but not numbers, of resident alveolar macrophages and monocytes were found in the lungs of castrated mice, (Fig. 2D, E). This castration-induced cell decrease was also at least partially reversed upon testosterone treatment (Fig. 2D, E). Androgen level modulation exerted no significant impact on the remaining examined innate and adaptive lung-resident immune cell populations (Fig. 2F, H, I, Supplementary Fig. 5). Regarding the intravascular circulating immunity, the impact of androgens was obvious on B cells, since circulating B cells followed the same pattern with their lung-resident counterpart (Supplementary Fig. 9).

### Alterations in lung-resident immunity upon ovariectomy

**Fig. 3:**
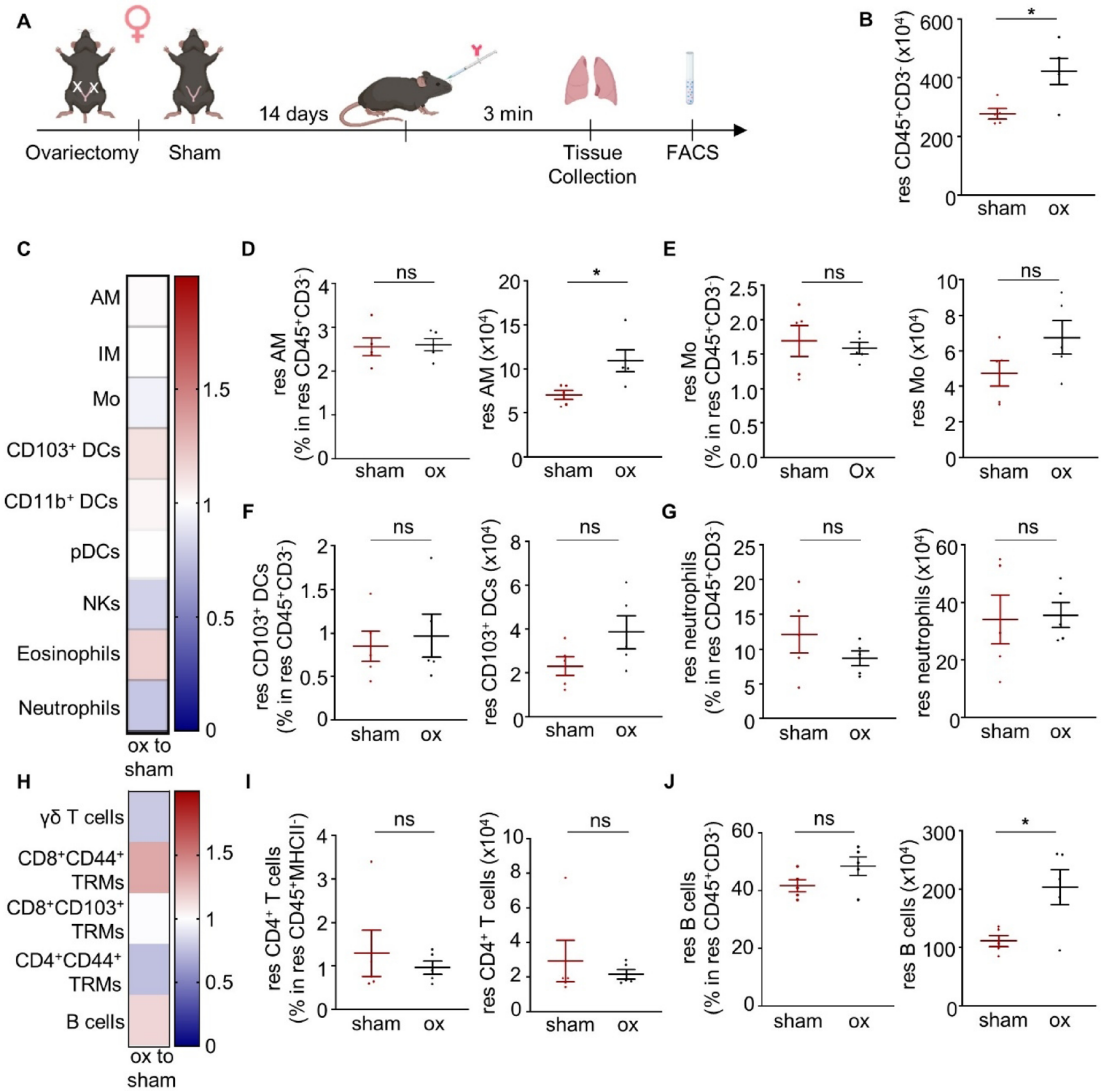
Alterations in lung-resident immunity upon ovariectomy. **(A)** Schematic view of the experimental setup (created with Biorender). **(B)** Number of resident CD45^+^CD3^-^ cells in the lungs of ovariectomised (ox) and sham operated female mice. **(C)** Heatmap depicting the frequency of innate immune cells residing in the lungs of female ovariectomised (ox) mice normalised to the frequency of innate immune cells found in the lungs of female sham operated (sham) mice. **(D**–**G)** Frequency in resident CD45^+^CD3^−^ cells and number of resident **(D)** alveolar macrophages, **(E)** monocytes, **(F)** CD103^+^ DCs, and **(G)** neutrophils. (**H)** Heatmap depicting the frequency of adaptive immune cells residing in the lungs of ovariectomized mice normalised to the frequency of adaptive immune cells residing in the lungs of sham operated female mice. **(I)** Frequency in resident CD45^+^MHCII^−^ cells and number of resident CD4^+^ T cells. **(J)** Frequency in resident CD45^+^CD3^−^ cells and number of resident B cells. *n* = 5 mice per group. Each experiment was repeated twice. Data are shown as mean ± SEM. *: *p* ≤ 0.05, as assessed by Mann-Whitney test. Non-significant differences (*p* > 0.05) are stated as ns. res: resident; AM: alveolar macrophages; IM: interstitial macrophages; Mo: monocytes; DCs: dendritic cells; pDCs: plasmacytoid DCs; NKs: natural killer cells; TRMs: tissue-resident memory T cells.

To investigate the effect of estrogens on lung-resident immunity, 12-week-old female C57BL/6 mice underwent ovariectomy and their lung-resident immunity was assessed and compared with the one of sham operated control female mice 14 days post operation (Fig. 3A). Of note, while ovariectomy did not impact the frequencies of lung-resident immune cell populations, higher number of total CD45 + resident cells, alveolar macrophages and B cells were found in the lungs of ovariectomized mice (Fig. 3B–J, Supplementary Fig. 6).

### Testosterone administration affects lung-resident immunity of female mice

**Fig. 4:**
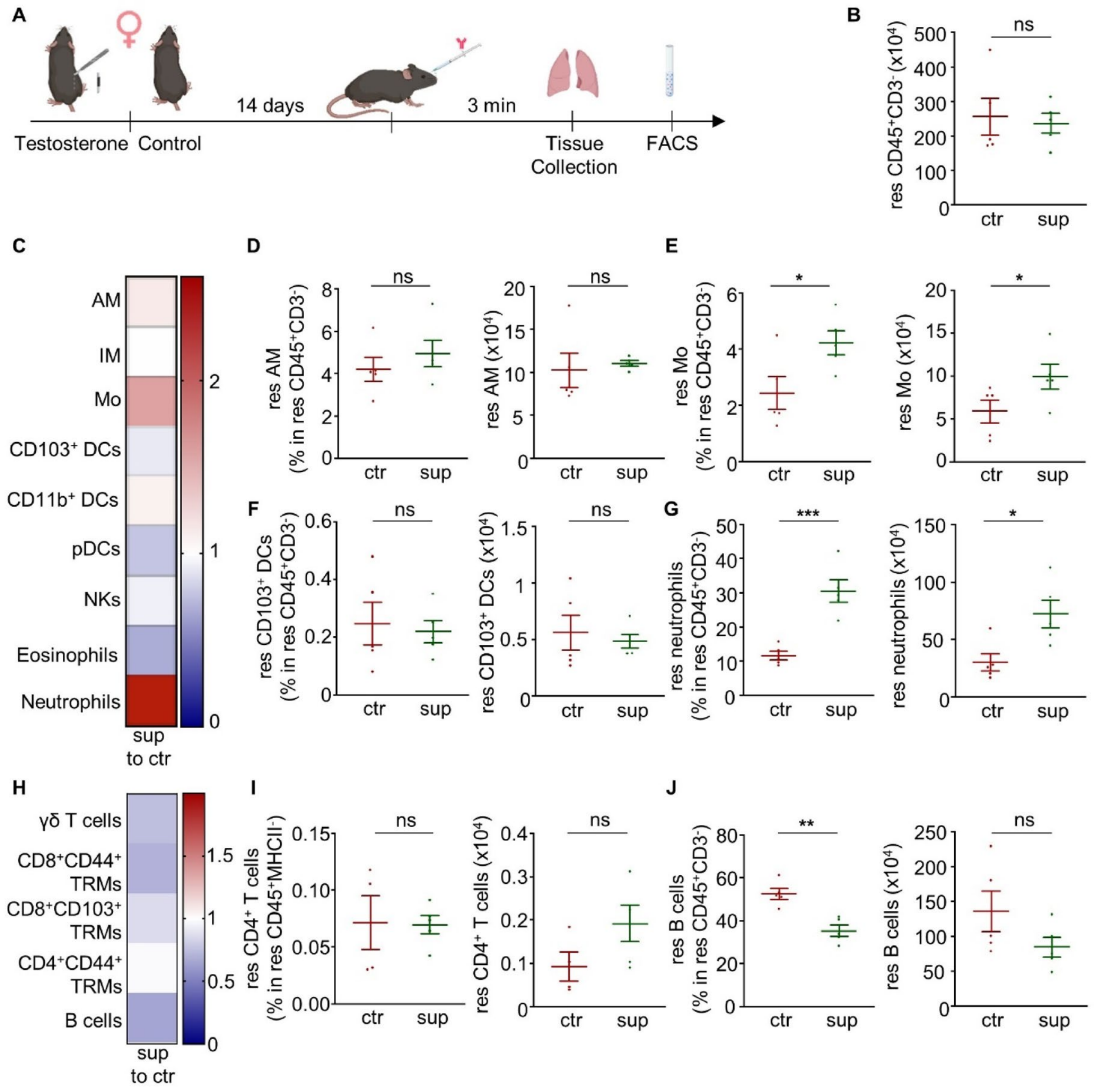
Testosterone supplementation affects lung-resident immunity of female mice. **(A)** Schematic view of the experimental setup (created with Biorender). **(B)** Number of resident CD45^+^CD3^-^ cells in the lungs of female testosterone-treated mice (sup) and control female mice (ctr). **(C)** Heatmap depicting the frequency of innate immune cells residing in the lungs of female testosterone-treated mice normalised to the respective frequencies found in the lungs of control female mice. **(D**–**G)** Frequency in resident CD45^+^CD3^−^ cells and number of resident **(D)** alveolar macrophages, **(E)** monocytes, **(F)** CD103^+^ DCs, and **(G)** neutrophils. **(H)** Heatmap depicting the frequency of adaptive immune cells residing in the lungs of testosterone-treated normalised to the frequency of adaptive immune cells residing in the lungs of control female mice. **(I)** Frequency in resident CD45^+^MHCII^−^ cells and number of resident CD4^+^ T cells. **(N)** Frequency in resident CD45^+^CD3^−^ cells and number of resident B cells. *n* = 5 mice per group. Each experiment was repeated twice. Data are shown as mean ± SEM. *: *p *≤ 0.05, **: *p* ≤ 0.01, ***: *p* ≤ 0.001, as assessed by Mann-Whitney test. Non-significant differences (*p* > 0.05) are stated as ns. res: resident; AM: alveolar macrophages; IM: interstitial macrophages; Mo: monocytes; DCs: dendritic cells; pDCs: plasmacytoid DCs; NKs: natural killer cells; TRMs: tissue-resident memory T cells.

Since testosterone manipulation strongly affected lung-resident immunity in male mice, we next administered testosterone also to female mice to examine the effect of such a supplementation on lung-resident immunity in this context. Testosterone implants were placed in adult C57BL/6 female mice and their lung-resident immunity was characterized 14 days after the implantation (Fig. 4A). Similar to our observations in testosterone-treated male castrated mice, testosterone supplementation affected several lung-resident immune cell populations (Fig. 4B–J, Supplementary Fig. 7). Specifically, a profound increase of lung-residing monocytes and neutrophils, both in numbers and frequencies, was observed in testosterone-treated female mice, in agreement to our findings in testosterone-supplemented castrated male mice (Fig. 4E, G). On the other hand, most examined lung-resident adaptive immune cell populations were reduced upon testosterone supplementation, with the most profound reduction in frequency seen in the case of lung-residing B cells (Fig. 4J). Circulating neutrophils were similarly affected following testosterone supplementation (Supplementary Fig. 9).

### Using sex-mismatched orthotopic lung transplantation to study sex specificity in lung-resident immunity

**Fig. 5:**
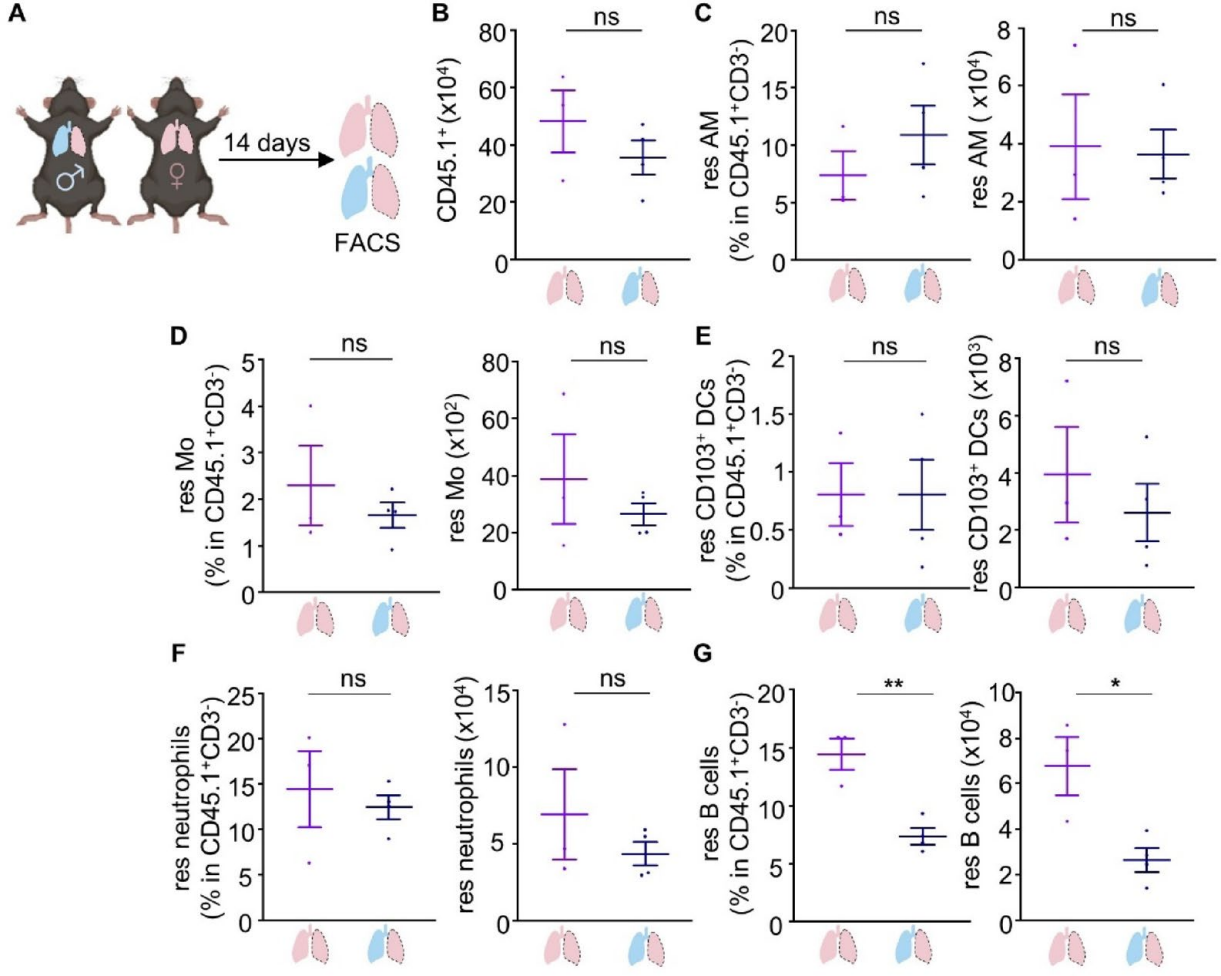
Lung-resident immunity upon sex-mismatched orthotopic lung transplantation in mice. **(A)** Schematic view of the experimental setup (created with Biorender). Lungs isolated from female donors (shown in pink surrounded by dashed lines) were transplanted into male or female recipients. **(B)** Number of resident CD45.1^+^ cells in the lungs of female donors (shown in pink surrounded by dashed lines) that were transplanted into male or female recipients. **(C**–**G)** Frequency in resident CD45^+^CD3^−^ cells and number of resident (CD45.1^+^) **(C)** alveolar macrophages, **(D)** monocytes, **(E)** CD103^+^ DCs, **(F)** neutrophils, and **(G)** B cells. *n* = 3–4 mice per group. Each experiment was repeated twice. Data are shown as mean ± SEM. *: *p* ≤ 0.05, **: *p* ≤ 0.01, as assessed by Mann-Whitney test. Non-significant differences (*p* > 0.05) are stated as ns. res: resident; AM: alveolar macrophages; Mo: monocytes; DCs: dendritic cells.

To conclusively determine the effect of a surrounding “male” macroenvironment on lung-resident immunity, including circulating male sex hormones and the recruitment of circulating immune cells into the lung, a state-of-the-art microsurgical technique for detection and characterization of tissue-resident immunity in mice was used, namely the orthotopic lung transplantation^12,17,18^. Specifically, the left lungs of female donor CD45.1^+^ mice were transplanted in male or female CD45.2^+^ recipients, with the latter serving as controls in this context (Fig. 5A). Lung-resident immunity in the transplanted lungs, identified as CD45.1^+^CD45.2^−^, was assessed 14 days after transplantation (Figs. 5B–G, Supplementary Fig. 8). Based on our previous findings indicating a profound effect of testosterone on lung-resident innate immunity and B cells with only minor alterations of T cell immunity, we mainly examined these immune cell components in the transplanted lungs.

Interestingly, female lungs transplanted into male recipients exhibited decreased lung-residing B cells compared to female lungs transplanted into female hosts (Fig. 5G). This result agrees with our observations upon castration of male and testosterone supplementation of male castrated and female mice and further highlights the crucial effect of testosterone on this lung-resident immune cell population.

## Discussion

Established sex differences characterize lung diseases^19^while data, mainly derived from epidemiological studies, suggest that sex hormones account for the observed sex bias in most cases^20^. Here, in order to shed light on the pathophysiological mechanisms underlying the sexual dimorphism of respiratory diseases, we investigated the sex differences in lung-resident immunity in healthy adult mice and the role of sex hormones in homeostatic conditions. The fact that most of the here examined lung-resident immune cell populations express sex hormone receptors, both in mouse and human lungs, supported our hypothesis^21^. Our results highlighted a key role of testosterone in shaping the observed sexual dimorphism, with the strongest effect observed on lung neutrophils and B cells.

Decreased lung-residing neutrophils and increased B cells were observed upon castration of male mice, differences which were reversed after testosterone supplementation of the castrated mice. Similar effects were also observed in testosterone-treated female mice or in “female” lungs transplanted into male recipients, with a decrease of lung-residing B cells and increase of neutrophils upon exposure to testosterone. Hence, lung-residing B cells and neutrophils seem to be either negatively or positively affected by androgens, respectively. These findings are in line with previous studies focusing on the circulating counterparts of B cells and neutrophils^22–24^. Neutrophils are reduced upon genetic ablation of the androgen receptor in mice, while androgens seem to trigger proliferation of neutrophil precursors via granulocyte colony-stimulating factor (G-CSF)-mediated signaling^25^. B cells populate the lung at early embryonic stages^26^ and even under homeostatic conditions, a diverse range of B cell subpopulations can be found in the lung^27^. The most studied and rapidly changing population are the resident memory B cells, which quickly expand after a local challenge^28^. In our case, since we examine homeostatic conditions, it is possible that other B cell subpopulations are particularly affected by hormone manipulation^29^. Other studies focusing on bone marrow, spleen and thymus have observed expansion of B cells after castration or androgen receptor depletion^23,30^and thus, a similar rapid expansion of the already residing in the lung B cells may take place in the absence of high androgen levels, even if the time period of 14 or 28 days might be short for some bone marrow-derived subsets. These findings are especially interesting in light of the already described regulation of circulating B cells by both the X chromosome and estrogens. B cell-mediated immune responses acquire sex-specific characteristics due to the escape of immune-related genes (e.g. TLR7) from X-inactivation, which leads to overreactive B cells in females compared to males^31–34^.

Although the effect of androgens on lung-resident immunity was here more profoundly studied, our data also demonstrate that female sex hormone deficiency due to ovariectomy increases the number of lung-resident B cells. Consistent with our findings, previous studies have shown that ovariectomy markedly increases B lymphocyte progenitors^35^, which might explain the effect observed here. On the other hand, peripheral B cell proliferation, activation, maturation, as well as survival and production of antigen-specific antibodies^36^ is enhanced by estrogens^37^. It should be noted that interpretation of findings deriving from the use of ovariectomy can be confusing, since the main female sex hormones, namely estrogens and progesterone, are highly produced in the ovaries in different phases of the menstrual cycle and can have opposite effects on the targeted immune cell populations^38^.

Although sex differences in immunity have been studied before^39^, most studies focused on the circulating immune compartment and ignored a potential sex specificity in tissue-resident immunity. To our knowledge, this is the first study employing established and state-of-the-art methods, such as orthotopic lung transplantation for tissue-resident immune cell detection, to thoroughly characterize the sexual dimorphism of lung-resident immunity^12,15^. Other studies focusing on sex differences in resident immunity of pleural and peritoneal cavities were conducted by Scotland et al.^40^, who performed a basic immunophenotyping to pinpoint F4/80+, T and B cells, and by Bain et al.^41^, who demonstrated a sexual dimorphism in peritoneal macrophages in both steady state and after *Streptococcus pneumoniae* infection. However, no approaches for distinguishing the circulating from the tissue-resident immune cell populations were used^12^. Additionally, the fact that circulating immune cell populations may present similar or even no sex differences, in comparison to their resident counterparts, as observed in our study, highlights the necessity of distinguishing and separately studying tissue-resident immunity. This point is highlighted here by the example of ILC2s: under steady-state conditions, total ILC2 levels (including both intravascular and extravascular compartments) are higher in females compared to males^16^, whereas when examining only the resident (extravascular) ILC2 population, this difference is no longer evident.

Regarding immune cell populations like neutrophils, which used to be considered as exclusively circulating cells, our findings are in line with other current studies, showing the presence and the important role of neutrophils in healthy tissues, and thus characterizing them in some cases as resident^42,43^. As evident following transplantation, donor-derived neutrophils can be still found in high frequencies in the transplanted lung two weeks after transplantation. Similarly, another population with tissue-residency properties appear to be pDCs, whose frequency increases in the transplanted lung 14 days post transplantation, in line with evidence suggesting a higher half-life of these cells compared to other DC subtypes^44^. Another point that needs to be clarified is that, although we employed naïve mice with no prior challenge exposure in this study, we were still able to isolate small TRM subsets. Multiple studies have demonstrated that T cells can be activated and expand into memory phenotypes, including TRM, as a result of interactions with commensal microbiota^45,46^. Therefore, the presence of TRM in naive mice does not imply any prior disease or infection in the mice used for this study. Interestingly, studies have shown that under homeostatic conditions, T cells with phenotypic and functional characteristics of memory cells can arise, despite lacking classical antigen specificity^47^.

Taken all findings together, one can assume that some of the sex differences found in lung-resident immunity can be attributed to sex hormones, while others cannot. Higher frequencies of resident macrophages in male lungs together with their reduction upon castration show that androgens might account for the sexual dimorphism of these lung-residing immune cell populations. However, testosterone supplementation in male castrated and female mice increased but did not fully restore the levels of lung-resident macrophages and monocytes, thereby suggesting that the effect of androgens might be counterbalanced by other factors in the female lung. Similarly, androgens seem to also influence lung-resident DCs, which were highly present in female naïve lungs and only slightly reduced upon testosterone supplementation. Although this finding could suggest a suppressive effect of androgens on these cells, castration also resulted in slight DC reduction thereby implying again that other factors besides sex hormones may affect lung-resident immunity. Such another factor affecting immune cells in a sex-specific way is the inactivation of the one X chromosome in females^7^.

Within the current study, we highlight the presence of sex differences in lung-resident immunity in healthy mice. Taking into account the important role of lung-resident immunity not only in the protection from lung diseases, but also in their pathogenesis^12,48^, such underlying sex differences may differentially determine disease susceptibility and course in males and females. Importantly, sex hormones seem to shape the observed sex differences by modulating the lung-resident immune cells and mounted responses, and could thus set the basis for novel personalized therapeutic approaches against respiratory immune diseases. Of note, since a clear effect of androgens on lung-resident immunity is here shown, one can assume that, due to changing sex hormone levels throughout the lifespan, lung-resident immunity composition is also changing. Such an age- and sex-specific variation of lung-resident immunity may explain the sex bias alterations seen across the lifespan in distinct respiratory immune diseases, such as asthma^7^. Further studies are needed to uncover the disease-specific implication of sex-specific lung-resident immunity and the related underlying mechanisms. Therapeutic approaches taking into account sex-specific disease susceptibility and pathogenesis could substantially improve life quality and disease prognosis.

### Limitations of the study

Our study demonstrates that testosterone may at least partially account for the sexual dimorphism seen in lung-resident immunity. However, our results also suggest that other factors, besides androgens, may contribute to the observed sex differences. Further studies are required to uncover such additional determinants of sex-specific lung-resident immunity in homeostatic conditions. Future work should also focus on the role of the here revealed immune sexual dimorphism in the sex bias of respiratory diseases and the related underlying mechanisms. Moreover, lung-resident immune cells were characterized 14 days after hormone manipulation procedures—a time period that might not be long enough to detect differences, particularly in tissue-resident memory T cells, which undergo prolonged differentiation and can persist in tissues for extended periods^49,50^. Regarding the observed differences, it remains elusive whether they derive from enhanced cell survival or accelerated proliferation, a question that could be addressed in future studies. Another limitation of the study and especially concerning the orthotopic lung transplantation model, is that CD45.2^+^ recipient-derived immune cells may have infiltrated the transplanted tissue within the two weeks following transplantation. However, we here focus on the CD45.1 + cells identified in the transplanted lung, which *per definition* come from the donor and are thus resident cells. Lastly, it must be taken into consideration that the cells referred to here as circulating are in fact intravascular cells within the lung, a population that may differ from immune cells isolated from peripheral blood.

## Methods

### Experimental animals

Adult male and female C57BL/6, CD45.1 and CD45.2 mice were purchased from Charles River and housed in the animal facility of the University Medical Centre Hamburg-Eppendorf in a 12-h light-dark circle with *ad libitum* access to food and water. This study was conducted and reported in accordance with the ARRIVE (Animal Research: Reporting of In Vivo Experiments) guidelines to ensure the transparent and comprehensive reporting of animal research. All animal studies were designed in accordance with the 3R (Replacement, Reduction, and Refinement) guidelines and respective European regulations^51^. All mice were in the age of 12–14 weeks old. The experimental procedures adhered to institutional guidelines and were approved by the respective German State authorities (Behörde für Gesundheit und Verbraucherschutz Hamburg; approval numbers, N20/29 and G19/75).

### In vivo CD45 cell staining

In order to distinguish CD45^+^ cells circulating in the bloodstream from CD45^+^ cells residing in the lung parenchyma, we injected intravenously 5 µg of an AF-700 conjugated anti-CD45 antibody (diluted in 100 µl PBS) into the right retro-orbital sinus of isoflurane-anaesthetized mice and euthanized them three minutes later by cervical dislocation^15^. For *in vitro* CD45 staining, a PE-Cy7 conjugated anti-CD45 antibody was used. Using flow cytometry, the lung-resident CD45^+^ cell population was later identified as PE-Cy7 positive and AF-700 negative.

### Castration and ovariectomy

Male and female mice were first put under anesthesia with isoflurane, then disinfected with 70% ethanol and shaved. In male mice, a midline incision in the scrotum was made to expose the testes. Next, the supplying vessels of the testes were carefully tied using 3.0 silk suture and the testes were removed. The incision was subsequently closed with 4.0 vicryl suture. Regarding the sham operated mice, the incision was closed immediately after exposing but not removing the testes.

In female mice, a single midline incision was made in each side of the abdomen. The peritoneum was opened and the ovaries were identified behind the intestine. Subsequently, the vessels and the oviduct were tied with 3.0 silk suture and the ovaries were removed. The peritoneum was sutured using 4.0 vicryl suture and the skin was next closed with metal clips. For the sham group, the skin and peritoneum were cut and subsequently closed as described above but the ovaries were not removed. 14 days after the operation, the mice were euthanized and tissue collection was performed as described below.

### Testosterone supplementation

In order to study the effect of testosterone, testosterone belma technologies^®^ implants were placed subcutaneously in male castrated or female mice. Mice were anesthetized with isoflurane, then shaved and a small incision was made on the top skin of their back. The implant was then placed in the incision and the skin was sutured. In control mice, the incision was closed without placing an implant. The implants release from 51.9 to 154.5 µg/24 h for plasma concentrations of 0.9–3.7 ng/ml. 14 days after the operation, the mice were euthanized and tissue collection was performed as described below.

### Orthotopic single left lung transplantation (LuTx)

Orthotopic LuTxes were performed as previously described^18^. Transplanted mice received no immunosuppression. CD45.1^+^ female mice were used as donors, and CD45.2^+^ mice (male or female, 2 groups) were used as recipients. By transplanting the CD45.1^+^ lung of the donor to the CD45.2^+^ recipient, tissue-resident immune cells are transplanted together with the organ in the recipient mouse, while the different congene expression in the transplanted organ and the periphery enables the discrimination between the tissue-resident and circulating immune compartment without the need for an *in vivo* CD45 cell staining. Briefly, donors were anesthetized using isoflurane. The pulmonary artery, bronchus, and pulmonary vein were carefully separated from one another with blunted forceps, prior to cuffing with 24-, 20-, and 22-gauge cuffs, respectively. The left lung graft was stored for less than 20 min before its implantation. The recipient mice were anesthetized using isoflurane, intubated and ventilated using a small-animal ventilator (UNO Apparatus) at a respiratory rate of 120 bpm and a tidal volume of 300 ml. The chest was opened on the left side between the third and fourth ribs and the native left lung was retracted with a clamp. The hilar structures were carefully separated from one another with blunted forceps. After arrest of the blood and air flow towards the left lung, the cuffed graft pulmonary artery, bronchus, and pulmonary vein were inserted into the recipient counterparts and ligated with 10 − 0 sutures. The native left lung was removed and the incision in the chest was closed with a 6 − 0 suture, after removing all potential air bubbles from the chest. The mice were extubated. After the operation, the recipient mice were allowed to recover at 30 °C overnight and received buprenorphine for 3 days. The mice were sacrificed 14 days post transplantation.

### Tissue collection

Mice were anaesthetized in an isoflurane chamber. Three minutes after the injection of the CD45^+^ antibody, described above, mice were sacrificed by cervical dislocation. A large incision was made from the lower abdomen up to the neck of the mouse. An incision of the left ventricle of the heart was conducted and then the lungs were perfused with 10 ml PBS via the right ventricle of the heart. The lungs were carefully collected, cleaned from all the surrounding tissues and placed in complete RPMI.

### Single cell isolation from mouse organs

To proceed with flow cytometry, single-cell suspension from the lungs was first generated. The collected lungs were minced and digested using 10 µl collagenase D (working concentration: 2 mg/ml) and 2 µl DNase I (10 U/µl) dissolved in 1x PBS. Samples were incubated at 37 °C for 30 min and then the digested lungs were passed through a 40-µm cell strainer and diluted in 40 ml 1% FCS dissolved in 1 x PBS. After centrifugation at 450 g for 8 min at 4 °C, the cell pellet was resuspended in 4 ml 40% Percoll solution and was slowly added on top of a 67% Percoll solution. After centrifugation at 400 g for 30 min at 20 °C, 1 ml of the middle phase containing the immune cells was taken and resuspended in 30 ml 1% FCS dissolved in 1 x PBS. After centrifugation at 450 g for 8 min at 4 °C, the cell pellet was diluted in 1 ml 1% FCS dissolved in 1 x PBS.

### Flow cytometry

The cell solution was separated in two equal parts (as two panels were applied) and transferred in flow cytometry tubes. Following blocking of unspecific binding using a rat anti-mouse CD16/CD32 Mouse Fragment Crystallizable Block (1:200; BD Bioscience) and normal rat serum (1:100; eBioscience, San Diego, CA), the cells were incubated with the respective antibodies for surface staining (Table 1). Data were acquired using a BD LSRFortessa II flow cytometer (BD Bioscience) and analysed using FlowJo v.10.8 Software (Treestar, Ashland, OR).

**Table 1:** Antibodies used in this study.

Conjugated fluorochrome	Antigen	Dilution	Company
AF700	CD45	1:400	BioLegend, San Diego, CA, USA
AF700	CD45.1	1:400	BioLegend, San Diego, CA, USA
AF488 (FITC)	Ly-6 g	1:400	BioLegend, San Diego, CA, USA
APC	CD11c	1:200	BD Bioscience, New Jersey, USA
APC	TCDγδ	1:200	eBioscience, San Diego, CA, USA
APC	Lineage	1:100	BD Bioscience, New Jersey, USA
APC-Cy7	CD11b	1:200	BD Bioscience, New Jersey, USA
APC-Cy7	CD44	1:400	BioLegend, San Diego, CA, USA
BUV 737	CD4	1:800	BD Bioscience, New Jersey, USA
BV421	CD19	1:400	BioLegend, San Diego, CA, USA
BV421	CD69	1:200	BioLegend, San Diego, CA, USA
BV421	IL-7Ra	1:100	BioLegend, San Diego, CA, USA
BV510	CD3	1:200	BioLegend, San Diego, CA, USA
BV510	CD19	1:400	BioLegend, San Diego, CA, USA
BV510	MHC II	1:600	BioLegend, San Diego, CA, USA
eFluor 506 (BV510)	Fixable viability dye	1:1000	eBioscience, San Diego, CA, USA
BV650	MHCII	1:800	BioLegend, San Diego, CA, USA
BV650	CD8	1:800	BioLegend, San Diego, CA, USA
BV711	NK-1.1	1:300	BioLegend, San Diego, CA, USA
BV785	CD103	1:100	BioLegend, San Diego, CA, USA
BV786	T-bet	1:100	BD Bioscience, New Jersey, USA
FITC	CD3	1:200	BD Bioscience, New Jersey, USA
AF488 (FITC)	GATA-3	1:100	BD Bioscience, New Jersey, USA
PE	Siglec-F	1:50	BD Bioscience, New Jersey, USA
PE-Texas Red	F4/80	1:200	Invitrogen Carlsbad, CA, USA
PE-Texas Red (PE-eFluor™ 610)	RORγτ	1:100	Invitrogen Carlsbad, CA, USA
PE-Cy7	CD45	1:400	BioLegend, San Diego, CA, USA
PE-Cy7	CD45.2	1:400	BioLegend, San Diego, CA, USA
PerCP-Cy5.5	Ly-6c	1:200	BioLegend, San Diego, CA, USA
PerCP-Cy5.5	CD62L	1:400	BioLegend, San Diego, CA, USA

### Statistical analysis

Animals were allocated to different groups by alternation. The results shown represent mean ± SEM. Comparisons between two groups were performed using t-test or Mann-Whitney test, depending on the normality of distribution. Comparisons among three groups were performed using One-Way ANOVA. P values were considered statistically significant when < 0.05 (*: *p* ≤ 0.05; **: *p* ≤ 0.01; ***: *p* ≤ 0.001; ****: *p* ≤ 0.0001). All statistical analyses were done and plots were created using GraphPad Prism versions 8.0 and 9.0.

## Supplementary Information

Below is the link to the electronic supplementary material.

Supplementary Material 1

Supplementary Material 2

Supplementary Material 3

Supplementary Material 4

Supplementary Material 5

Supplementary Material 6

Supplementary Material 7

Supplementary Material 8

Supplementary Material 9

Supplementary Material 10

## Data Availability

The data of this study are available from the corresponding author upon reasonable request.

## Abbreviations

AM: Alveolar macrophages
AR: Androgen receptor
CD: Cluster of Differentiation
COPD: Chronic obstructive pulmonary disease
DCs: Dendritic cells
EDTA: Ethylenediaminetetraacetic acid solution
FCS: Fetal calf serum
FSC: Forward scatter
G-CSF: Granulocyte colony-stimulating factor
ILCs: Innate lymphoid cells
IM: Interstitial macrophages
MHCII: Major histocompatibility complex class II
NK: Natural killer cells
NRS: Normal rat serum
pDCs: Plasmacytoid dendritic cells
PFA: Paraformaldehyde
SSC: Side scatter
TRMs: Tissue resident memory cells
WHO: World’s Health Organization
