# Supplementary figures and images for: Sex hormones regulate the sexual dimorphism of the lung resident immune milieu
Source: Sci Rep. 2025 Aug 31;15:32032. doi: 10.1038/s41598-025-15941-6 (PMC12399755; doi:10.1038/s41598-025-15941-6)

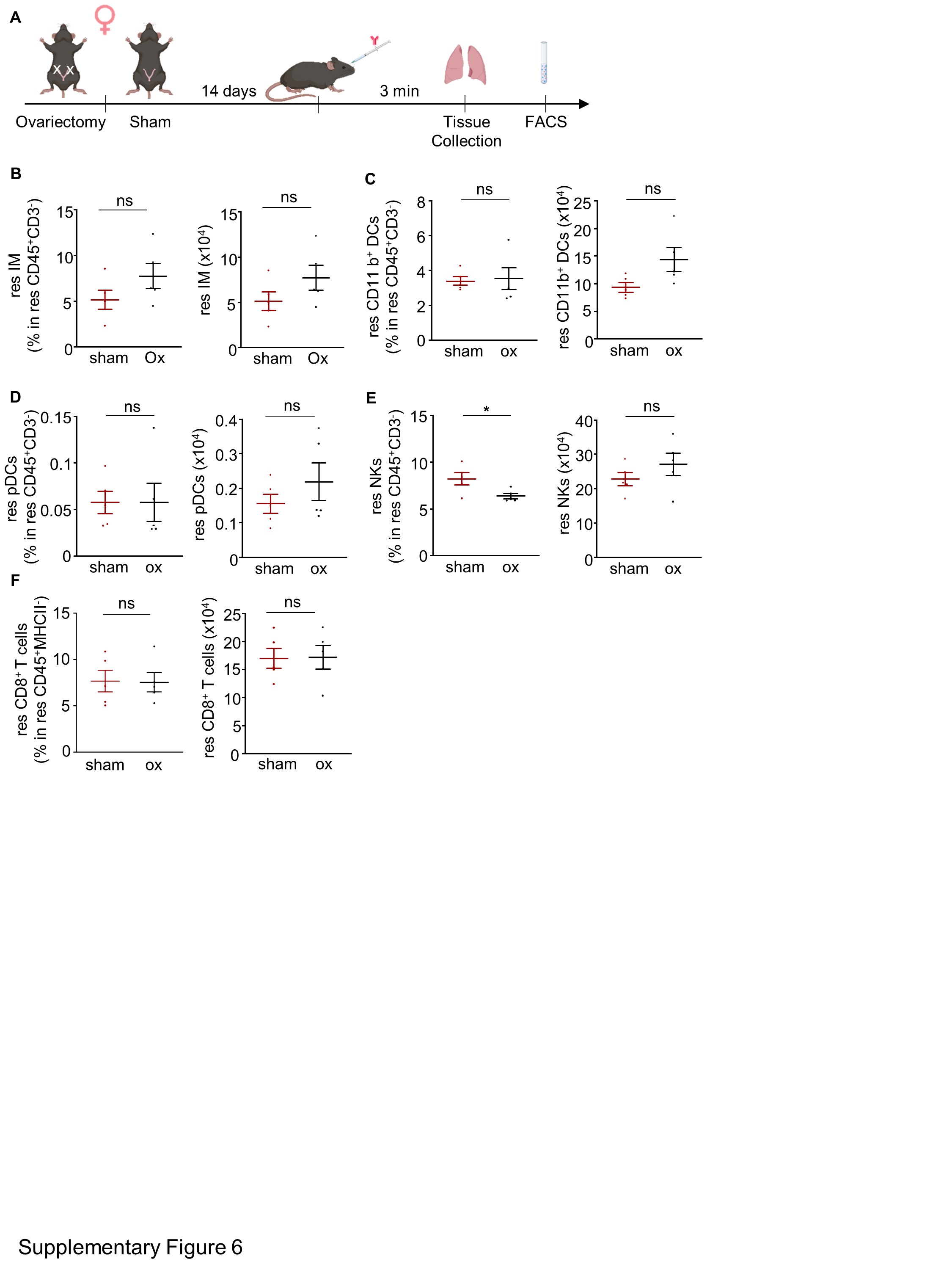

Supplement: Supplementary file 1 — Supplementary Material 1 [file 41598_2025_15941_MOESM1_ESM.jpg]

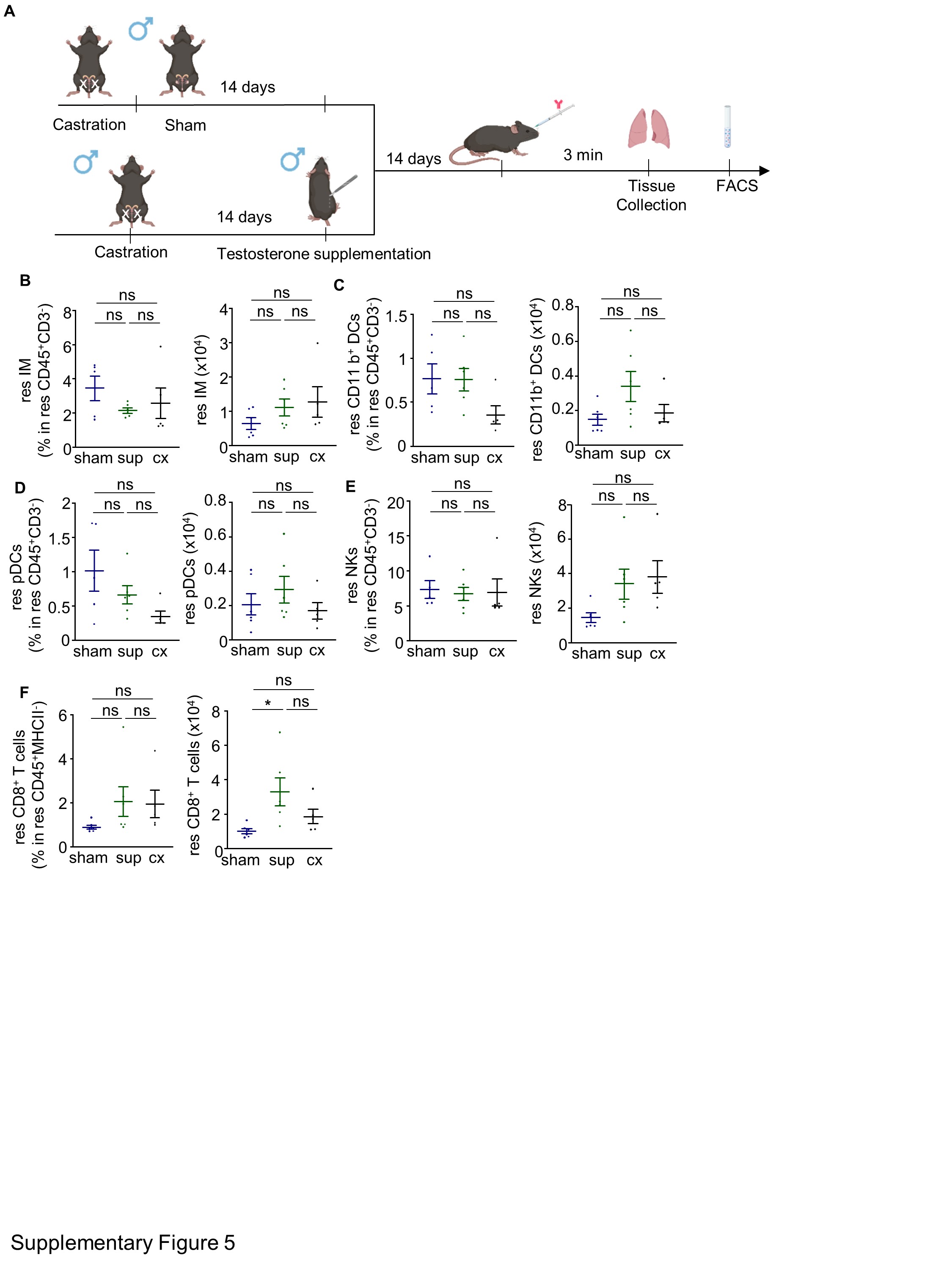

Supplement: Supplementary file 2 — Supplementary Material 2 [file 41598_2025_15941_MOESM2_ESM.jpg]

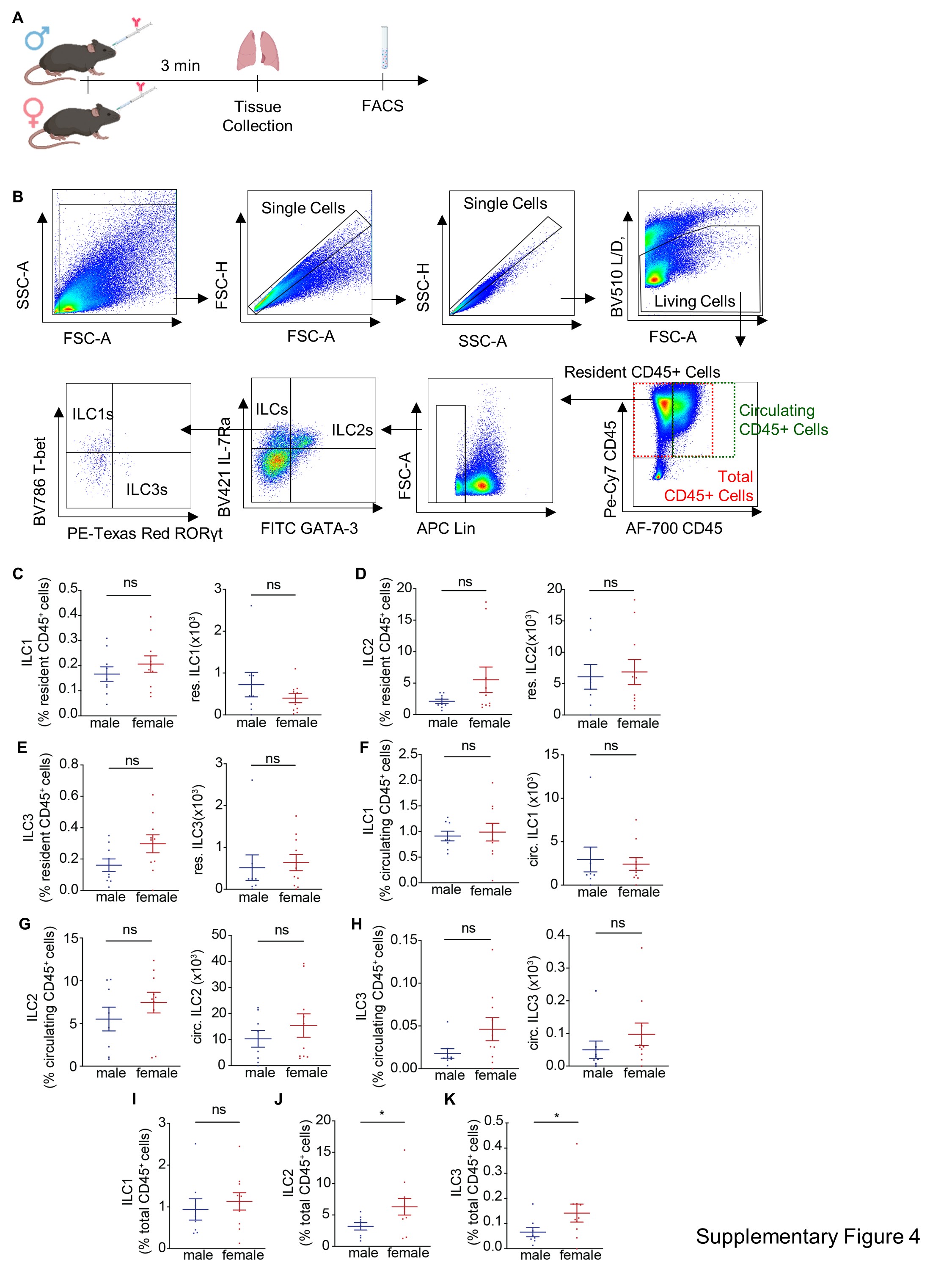

Supplement: Supplementary file 3 — Supplementary Material 3 [file 41598_2025_15941_MOESM3_ESM.jpg]

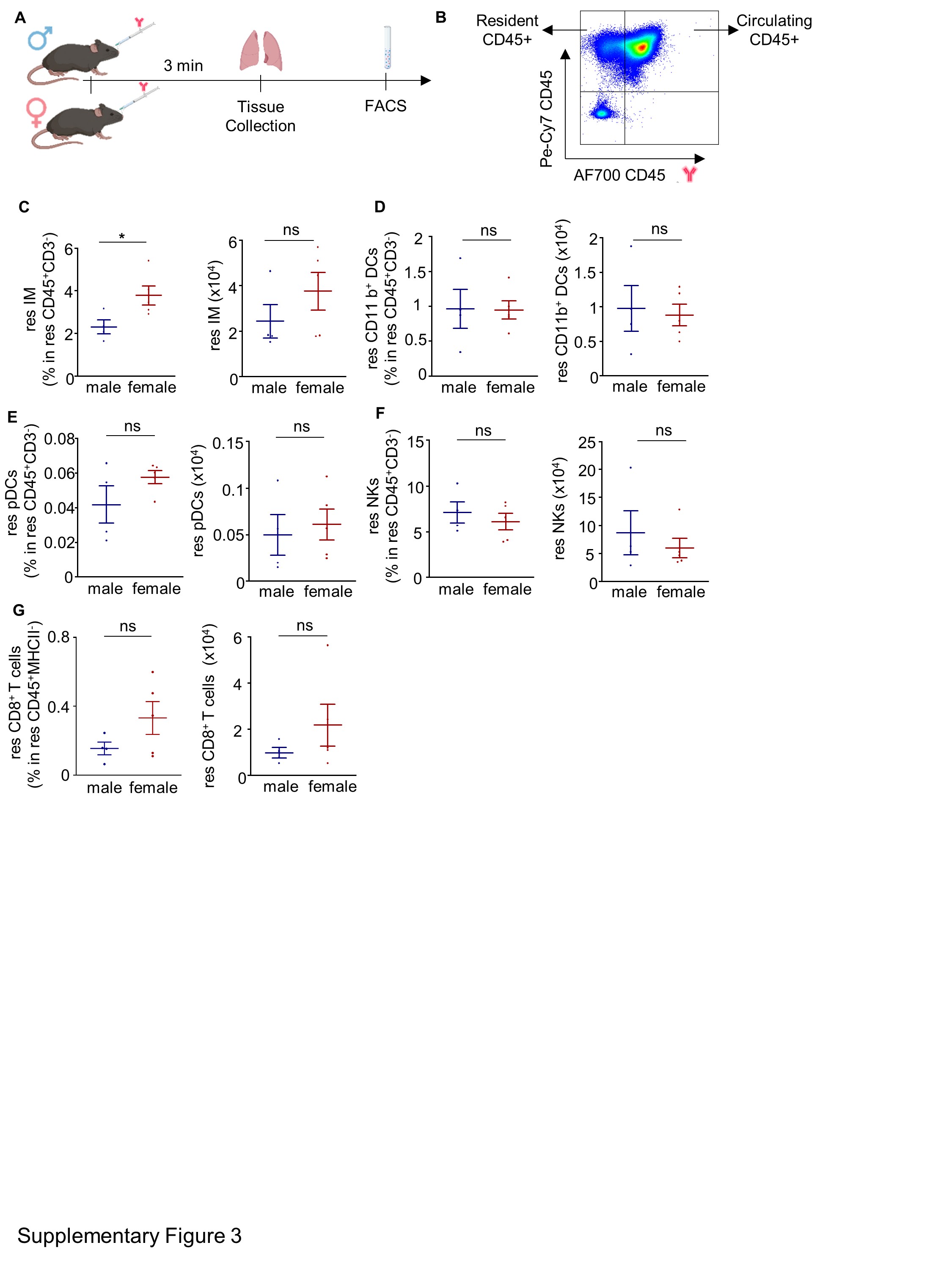

Supplement: Supplementary file 4 — Supplementary Material 4 [file 41598_2025_15941_MOESM4_ESM.jpg]

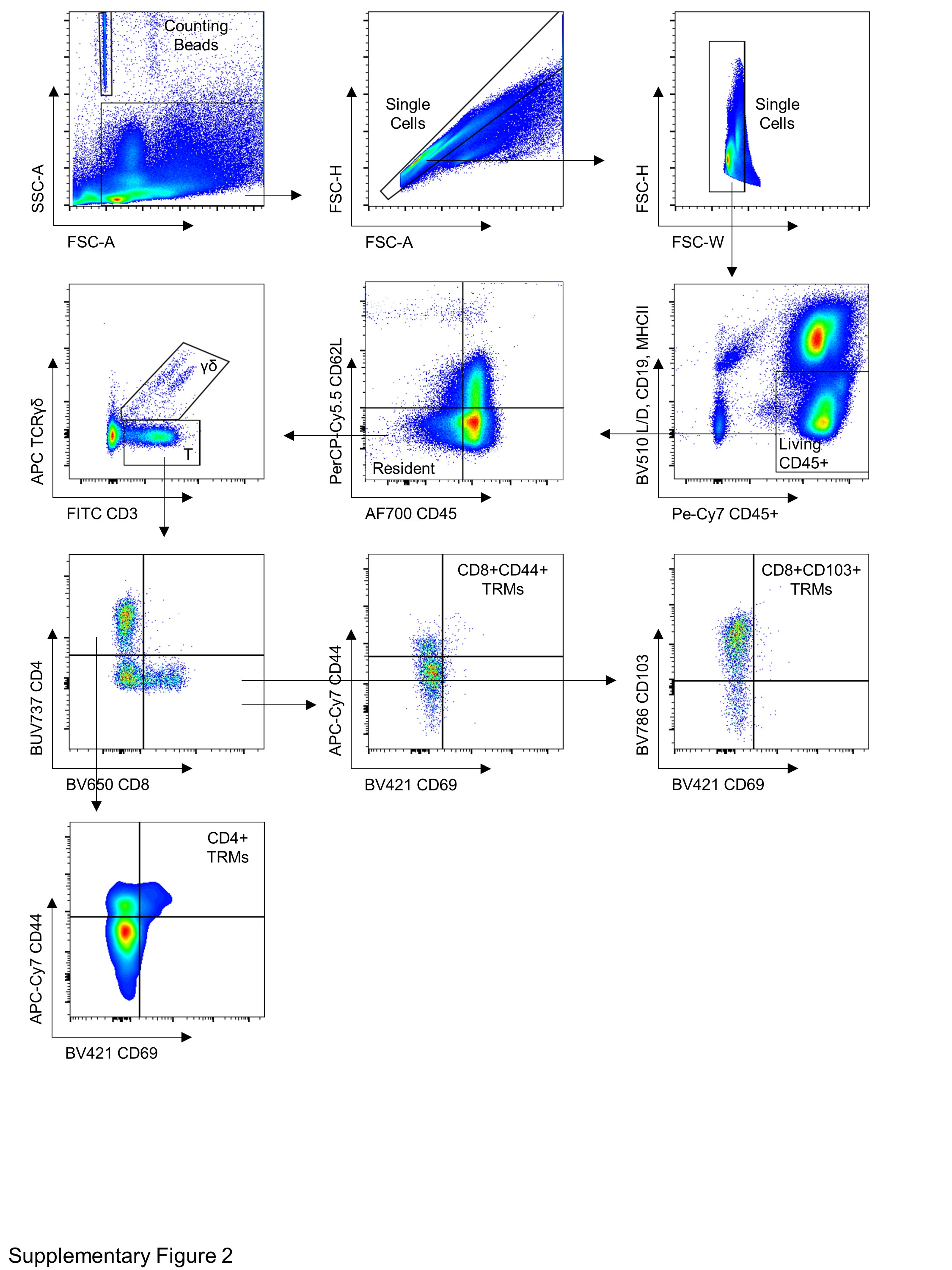

Supplement: Supplementary file 5 — Supplementary Material 5 [file 41598_2025_15941_MOESM5_ESM.jpg]

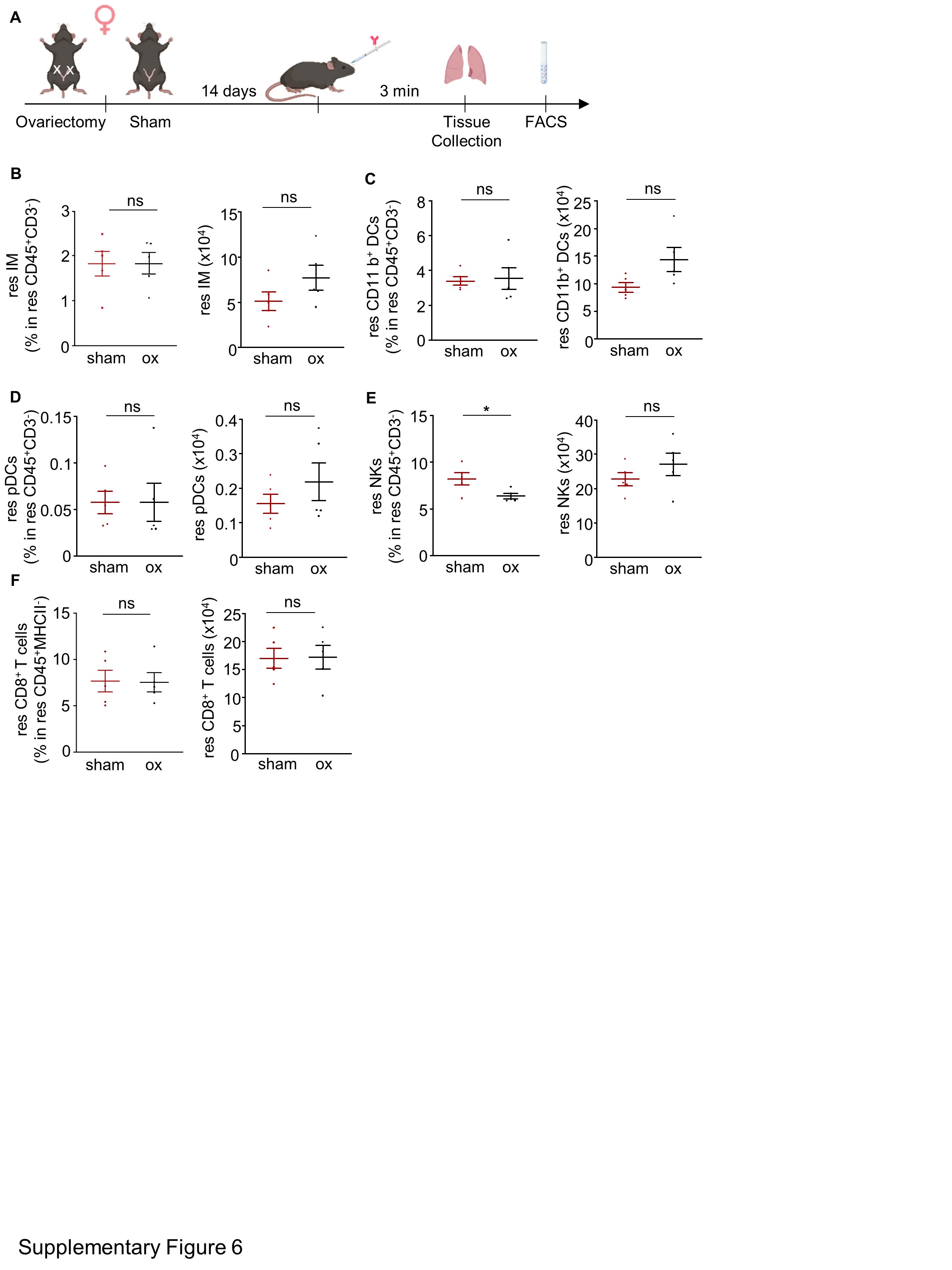

Supplement: Supplementary file 6 — Supplementary Material 6 [file 41598_2025_15941_MOESM6_ESM.jpg]

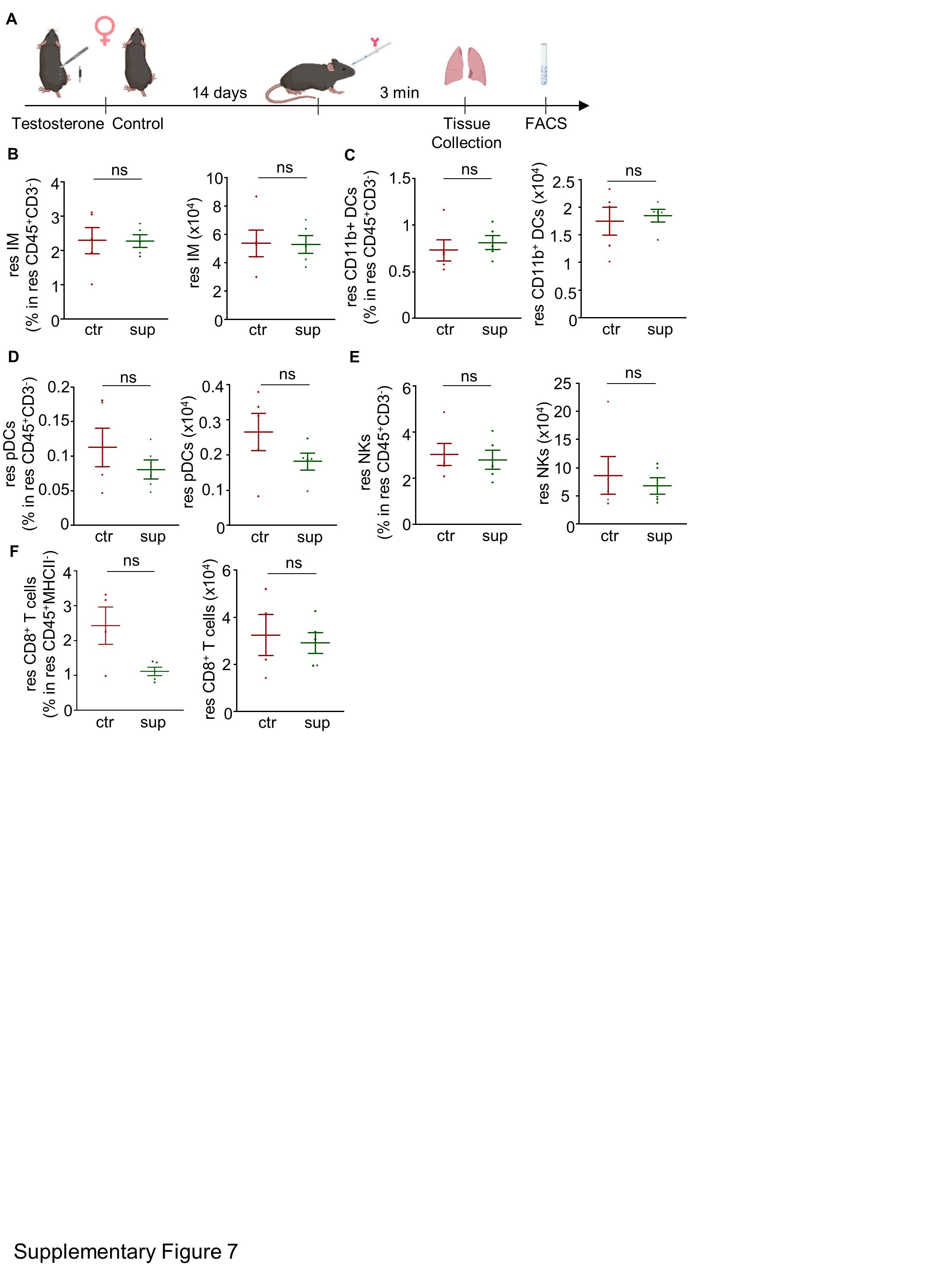

Supplement: Supplementary file 7 — Supplementary Material 7 [file 41598_2025_15941_MOESM7_ESM.jpg]

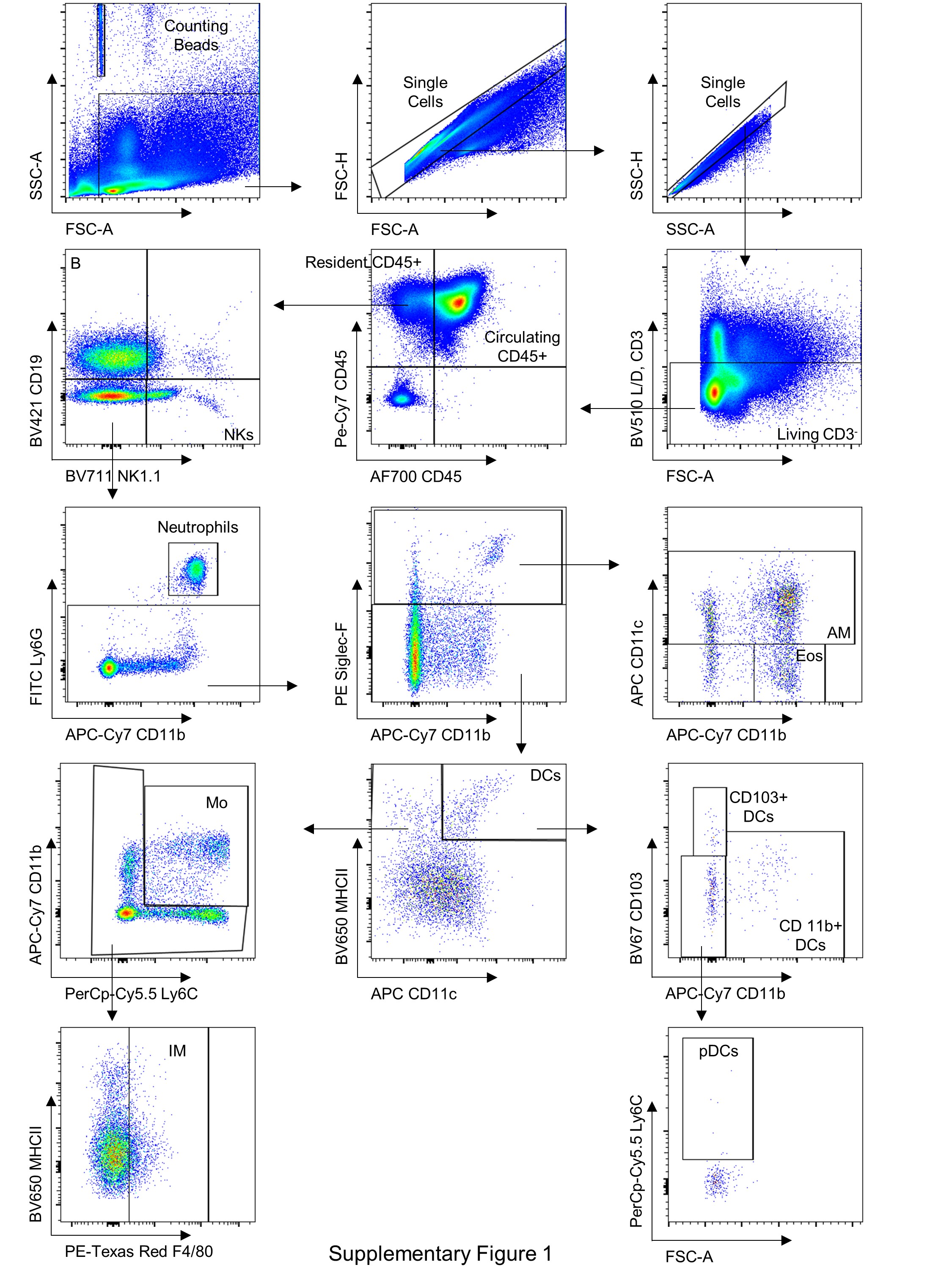

Supplement: Supplementary file 8 — Supplementary Material 8 [file 41598_2025_15941_MOESM8_ESM.jpg]

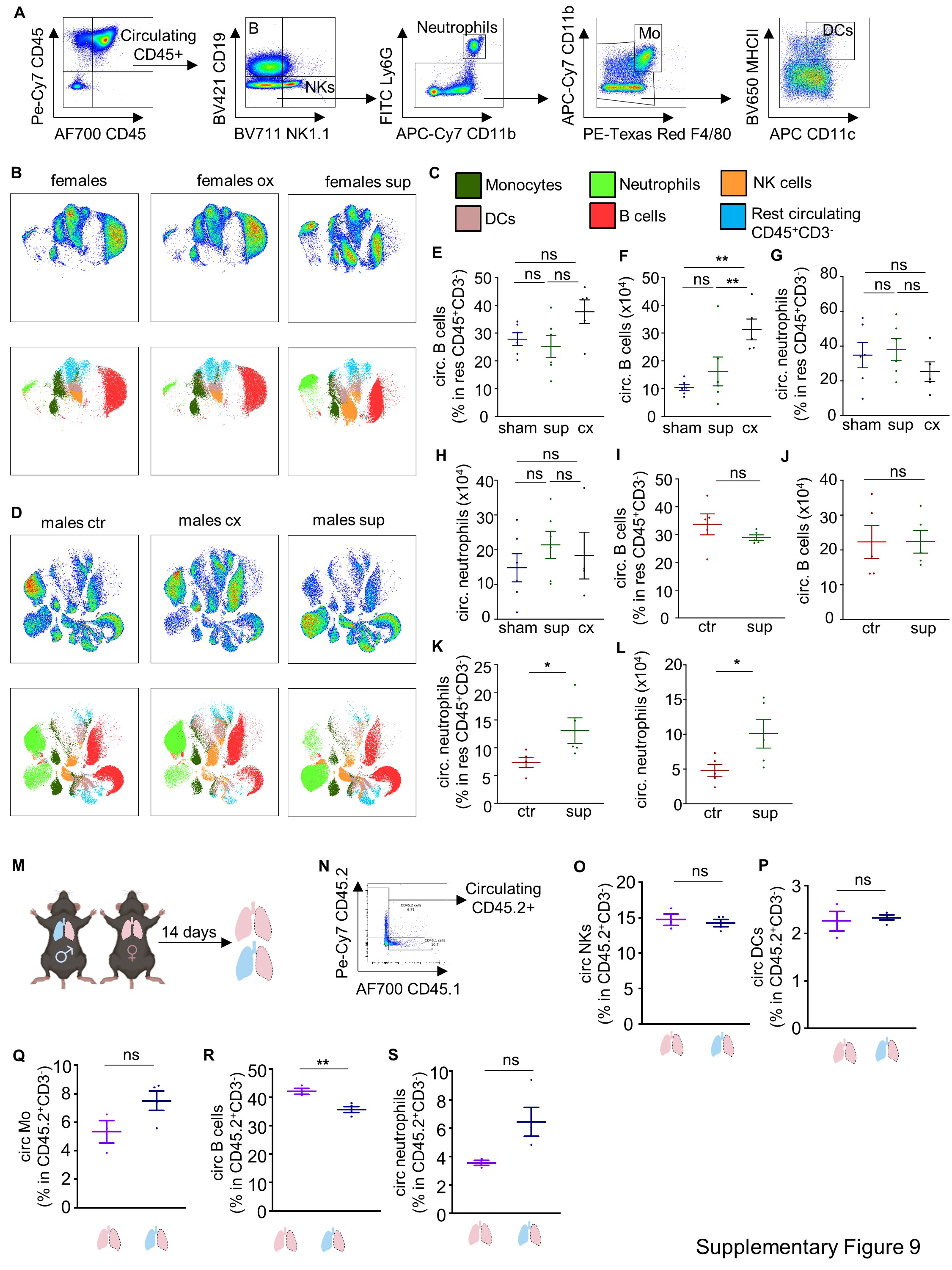

Supplement: Supplementary file 9 — Supplementary Material 9 [file 41598_2025_15941_MOESM9_ESM.jpg]
