# Supplementary material for: Sex hormones regulate the sexual dimorphism of the lung resident immune milieu
Source: Sci Rep. 2025 Aug 31;15:32032. doi: 10.1038/s41598-025-15941-6 (PMC12399755; doi:10.1038/s41598-025-15941-6)
Supplement: Supplementary file 10 — Supplementary Material 10 [file 41598_2025_15941_MOESM10_ESM.docx]

**Sex hormones regulate the sexual dimorphism of the lung resident immune milieu**

Ioannis Belios^1^, Tao Zhang^2^, Christopher Urbschat^1^, Jun Oh^3^, Wolfgang Jungraithmayr^4,5,6^, Samuel Huber^2,7^, Petra C. Arck^1,7^, Anastasios D. Giannou^2,7,8^, Dimitra E. Zazara*^1,3,7^

^1^Division for Experimental Feto-Maternal Medicine, Department of Obstetrics and Fetal Medicine, University Medical Center of Hamburg-Eppendorf (UKE), Germany

^2^Section of Molecular Immunology and Gastroenterology, I. Department of Medicine, UKE, Germany

^3^University Children's Hospital, UKE, Hamburg, Germany

^4^Division of Thoracic Surgery, Rostock University Medical Center, Rostock, Germany

^5^Department of Thoracic Surgery, University Hospital Zurich, Zurich, Switzerland

^6^Department of Thoracic Surgery, Medical Center-University of Freiburg, Faculty of Medicine, University of Freiburg, Freiburg, Germany

^7^Hamburg Center for Translational Immunology, UKE, Germany

^8^Department of General, Visceral and Thoracic Surgery, UKE, Germany

**Correspondence:**

Dimitra E. Zazara, M.Sc., M.D., PhD; Division for Experimental Feto-Maternal Medicine, Department of Obstetrics and Fetal Medicine and University Children's Hospital, University Medical Center Hamburg-Eppendorf, Martinistr. 52, 20246 Hamburg, Germany. Email: di.zazara@uke.de, Phone: +49 40 7410 58710.

**SUPPLEMENTARY FIGURE LEGENDS**

**Figure S1:** Gating strategy for lung-resident innate immunity

**Figure S2:** Gating strategy for lung-resident adaptive immunity

**Figure S3: (A)** Schematic view of the experimental setup (created with Biorender). **(B)** Flow cytometry-based distinction of lung-resident and circulating CD45^+^ cells. **(C-G)** Frequency in resident CD45^+^CD3^-^ cells and number of resident **(C)** interstitial macrophages, **(D)** CD11b^+^ DCs, **(E)** pDCs, **(F)** NK cells, and **(G)** CD8^+^ TRMs in male and female mouse lungs. n= 4-5 mice per group. Each experiment was repeated twice. Data are shown as mean ± SEM. *: p≤ 0.05, as assessed by Mann-Whitney test. Non-significant differences (p > 0.05) are stated as ns. res: resident; IM: interstitial macrophages; (p)DCs: (plasmacytoid) dendritic cells; NKs: natural killer cells; TRMs: tissue-resident memory T cells.

**Figure S4: (A)** Schematic view of the experimental setup (created with Biorender). **(B)** Representative gating strategy in order to analyse the ILCs in the lung. **(C-E)** Frequency in resident CD45^+^ cells and number of resident **(C)** ILC1s, **(D)** ILC2s, and **(E)** ILC3s in male and female mouse lungs. **(F-H)** Frequency in circulating CD45^+^ cells and numbers of circulating **(F)** ILC1s, **(G)** ILC2s, and **(H)** ILC3s isolated from male and female mouse lungs. **(I-K)** Frequency in total CD45^+^ cells of total **(I)** ILC1s, **(J)** ILC2s, and **(K)** ILC3s isolated from male and female mouse lungs. n= 8-11 mice per group. Each experiment was repeated twice. Data are shown as mean ± SEM. *: p≤ 0.05, as assessed by Mann-Whitney test. Non-significant differences (p > 0.05) are stated as ns, as assessed by Mann-Whitney test. res: resident; ILC: Innate Lymphoid Cells.

**Figure S5: (A)** Schematic view of the experimental setup (created with Biorender). **(B-F)** Frequency in resident CD45^+^CD3^-^ cells and number of resident **(B)** interstitial macrophages, **(C)** CD11b^+^ DCs, **(D)** pDCs, **(E)** NK cells, and **(F)** CD8^+^ TRMs in the lungs of sham operated, castrated and then supplemented with testosterone (sup), as well as castrated (cx) male mice. n= 5-6 mice per group. Each experiment was repeated twice. Data are shown as mean ± SEM. *: p≤ 0.05, as assessed by Mann-Whitney test. Non-significant differences (p > 0.05) are stated as ns. res: resident; IM: interstitial macrophages; (p)DCs: (plasmacytoid) dendritic cells; NKs: natural killer cells; TRMs: tissue-resident memory T cells.

**Figure S6: (A)** Schematic view of the experimental setup (created with Biorender). **(B-F)** Frequency in resident CD45^+^CD3^-^ cells and number of resident **(B)** interstitial macrophages, **(C)** CD11b^+^ DCs, **(D)** pDCs, **(E)** NK cells, **(F)** CD8^+^ TRMs in the lungs of ovariectomised (ox) and sham operated female mice. n= 5 mice per group. Each experiment was repeated twice. Data are shown as mean ± SEM. *: p≤ 0.05, as assessed by Mann-Whitney test. Non-significant differences (p > 0.05) are stated as ns. res: resident; IM: interstitial macrophages; (p)DCs: (plasmacytoid) dendritic cells; NKs: natural killer cells; TRMs: tissue-resident memory T cells.

**Figure S7: (A)** Schematic view of the experimental setup (created with Biorender). **(B-F)** Frequency in resident CD45^+^CD3^-^ cells and number of resident **(B)** interstitial macrophages, **(C)** CD11b^+^ DCs, **(D)** pDCs, **(E)** NK cells, and **(F)** CD8^+^ TRMs in the lungs of female testosterone-treated mice (sup) and control female mice (ctr). n= 5 mice per group. Each experiment was repeated twice. Data are shown as mean ± SEM. Non-significant differences (p > 0.05) are stated as ns, as assessed by Mann-Whitney test. res: resident; IM: interstitial macrophages; (p)DCs: (plasmacytoid) dendritic cells; NKs: natural killer cells; TRMs: tissue-resident memory T cells.

**Figure S8: (A)** Schematic view of the experimental setup (created with Biorender). Lungs isolated from female donors (shown in pink surrounded by dashed lines) were transplanted into male or female recipients. **(B-E)** Frequency in resident CD45^+^CD3^-^ cells and number of resident (CD45.1^+^) **(B)** interstitial macrophages, **(C)** CD11b^+^ DCs, **(D)** pDCs, and **(E)** NK cells. n= 3-4 mice per group. Each experiment was repeated twice. Data are shown as mean ± SEM. Non-significant differences (p > 0.05, as assessed by Mann-Whitney test) are stated as ns. res: resident; IM: interstitial macrophages; DCs: dendritic cells; pDCs: plasmacytoid DCs; NKs: natural killer cells.

**Figure S9: (A)** Gating strategy for analysing the circulating CD45^+^ cells of all the experiments. **(B)** UMAPs indicating density (upper row in each individual square) and cluster identity (lower raw in each individual square) of the circulating CD45^+^ cells, isolated from lung, for the experiments conducted in female mice. **(C)** The identity of the clusters shown in the UMAPs. **(D)** UMAPs indicating density (upper row in each individual square) and cluster identity (lower raw in each individual square) of the circulating CD45^+^ cells, isolated from lung, for the experiments conducted in male mice. **(E-H)** Frequency in circulating CD45^+^CD3^-^ cells and number of circulating **(E-F)** B cells, and **(G-H)** neutrophils isolated from the lungs of sham operated, castrated and then supplemented with testosterone (sup), as well as castrated (cx) male mice. **(I-L)** Frequency in circulating CD45^+^CD3^-^ cells and number of circulating **(I-J)** B cells, and **(K-L)** neutrophils isolated from the lungs of female testosterone-treated mice (sup) and control female mice (ctr). **(M)** Schematic view of the experimental setup (created with Biorender). Lungs isolated from female donors (shown in pink surrounded by dashed lines) were transplanted into male or female recipients. **(N)** Flow cytometry-based distinction of donor (resident) CD45.1^+^ and recipient (circulating) CD45.2^+^ cells. **(O-S)** Frequency of recipient (circulating), **(O)** NK cells, **(P)** dendritic cells, **(Q)** monocytes, **(R)** B cells, and **(S)** neutrophils in recipient (circulating) CD45.2 cells in the donor lung. n= 3-6 mice per group. Each experiment was repeated twice. Data are shown as mean ± SEM. *: p≤ 0.05, **: p≤ 0.01, as assessed by one-way ANOVA with multiple comparisons (E-H) or Mann-Whitney test (I-L, O-S), as appropriate. Non-significant differences (p > 0.05) are stated as ns. DCs: dendritic cells; NKs: natural killer cells; circ: circulating.
